# COVID-19 Incidence and Mortality Among Unvaccinated and Vaccinated Persons Aged ≥12 Years by Receipt of Bivalent Booster Doses and Time Since Vaccination — 24 U.S. Jurisdictions, October 3, 2021–December 24, 2022

**DOI:** 10.15585/mmwr.mm7206a3

**Published:** 2023-02-10

**Authors:** Amelia G. Johnson, Lauren Linde, Akilah R. Ali, Allison DeSantis, Minchan Shi, Carolyn Adam, Brandy Armstrong, Brett Armstrong, Madison Asbell, Steven Auche, Nagla S. Bayoumi, Boudu Bingay, Melisse Chasse, Scott Christofferson, Michael Cima, Kevin Cueto, Spencer Cunningham, Janelle Delgadillo, Vajeera Dorabawila, Cherie Drenzek, Brandi Dupervil, Tonji Durant, Aaron Fleischauer, Ross Hamilton, Pauline Harrington, Liam Hicks, Jeffrey D. Hodis, Dina Hoefer, Sam Horrocks, Mikhail Hoskins, Sofia Husain, L. Amanda Ingram, Amanda Jara, Amanda Jones, F. N. U. Kanishka, Ramandeep Kaur, Saadiah I. Khan, Samantha Kirkendall, Priscilla Lauro, Shelby Lyons, Joshua Mansfield, Amanda Markelz, John Masarik, Donald McCormick, Erica Mendoza, Keeley J. Morris, Enaholo Omoike, Komal Patel, Melissa A. Pike, Tamara Pilishvili, Kevin Praetorius, Isaiah G. Reed, Rachel L. Severson, Nekabari Sigalo, Emma Stanislawski, Sarah Stich, Buddhi P. Tilakaratne, Kathryn A. Turner, Caleb Wiedeman, Allison Zaldivar, Benjamin J. Silk, Heather M. Scobie

**Affiliations:** ^1^National Center for Immunization and Respiratory Diseases, CDC; ^2^CDC COVID-19 Emergency Response Team; ^3^Kentucky Department for Public Health; ^4^CDC Foundation, Atlanta, Georgia; ^5^West Virginia Department of Health and Human Resources; ^6^Indiana Department of Health; ^7^New Jersey Department of Health; ^8^Massachusetts Department of Public Health; ^9^Tennessee Department of Health; ^10^Utah Department of Health and Human Services; ^11^Arkansas Department of Health; ^12^Nebraska Department of Health and Human Services; ^13^New York State Department of Health; ^14^Georgia Department of Public Health; ^15^North Carolina Department of Health and Human Services; ^16^Booz Allen Hamilton, McLean, Virginia; ^17^Michigan Department of Health and Human Services; ^18^Arizona Department of Health Services; ^19^Washington State Department of Health; ^20^Alabama Department of Public Health; ^21^Center for Surveillance, Epidemiology and Laboratory Science, CDC; ^22^Idaho Department of Health and Welfare; ^23^Louisiana Department of Health; ^24^Minnesota Department of Health; ^25^District of Columbia Department of Health; ^26^Texas Department of State Health Services; ^27^Colorado Department of Public Health and Environment; ^28^New Mexico Department of Health; ^29^Kansas Department of Health and Environment.

On September 1, 2022, CDC recommended an updated (bivalent) COVID-19 vaccine booster to help restore waning protection conferred by previous vaccination and broaden protection against emerging variants for persons aged ≥12 years (subsequently extended to persons aged ≥6 months).* To assess the impact of original (monovalent) COVID-19 vaccines and bivalent boosters, case and mortality rate ratios (RRs) were estimated comparing unvaccinated and vaccinated persons aged ≥12 years by overall receipt of and by time since booster vaccination (monovalent or bivalent) during Delta variant and Omicron sublineage (BA.1, BA.2, early BA.4/BA.5, and late BA.4/BA.5) predominance.^†^ During the late BA.4/BA.5 period, unvaccinated persons had higher COVID-19 mortality and infection rates than persons receiving bivalent doses (mortality RR = 14.1 and infection RR = 2.8) and to a lesser extent persons vaccinated with only monovalent doses (mortality RR = 5.4 and infection RR = 2.5). Among older adults, mortality rates among unvaccinated persons were significantly higher than among those who had received a bivalent booster (65–79 years, RR = 23.7 and ≥80 years; 10.3) or a monovalent booster (65–79 years, 8.3 and ≥80 years; 4.2). In a second analysis stratified by time since booster vaccination, there was a progressive decline from the Delta period (RR = 50.7) to the early BA.4/BA.5 period (7.4) in relative COVID-19 mortality rates among unvaccinated persons compared with persons receiving who had received a monovalent booster within 2 weeks–2 months. During the early BA.4/BA.5 period, declines in relative mortality rates were observed at 6–8 (RR = 4.6), 9–11 (4.5), and ≥12 (2.5) months after receiving a monovalent booster. In contrast, bivalent boosters received during the preceding 2 weeks–2 months improved protection against death (RR = 15.2) during the late BA.4/BA.5 period. In both analyses, when compared with unvaccinated persons, persons who had received bivalent boosters were provided additional protection against death over monovalent doses or monovalent boosters. Restored protection was highest in older adults. All persons should stay up to date with COVID-19 vaccination, including receipt of a bivalent booster by eligible persons, to reduce the risk for severe COVID-19.

Previous reports on COVID-19 vaccine impact indicated that protection against infection and, to a lesser degree, severe illness, declined with waning of vaccine-induced immunity and emergence of the SARS-CoV-2 Delta and Omicron variants^§^ (*1*–*4*). After Omicron (BA.1) became predominant in the United States in late December 2021, Omicron sublineages BA.2, BA.4, and BA.5 circulated at high prevalence; BA.4 and BA.5-related variants constituted 78% of circulating lineages by December 24, 2022. Food and Drug Administration (FDA)–authorized bivalent boosters, which include an additional Omicron BA.4/BA.5 spike component, have been shown to enhance protection against infection and medically attended illness (*5*–*7*). 

Weekly counts of COVID-19 cases (October 3, 2021–December 24, 2022) and associated deaths (October 3, 2021–December 3, 2022) by primary series vaccination and booster status, including bivalent boosters (the week starting September 18, 2022), were reported from 24 jurisdictions^¶^ that routinely link case surveillance data to immunization registries (vaccinations) and vital registration databases (deaths). Accounting for case and death reporting lags (2 weeks and 5 weeks, respectively) permitted more complete reporting, data linkage, and mortality ascertainment. Standardized definitions were used for COVID-19 cases** and COVID-19–associated deaths^††^ by vaccination status^§§^ with specimen collection dates used as reference dates; vaccinated persons who did not complete a primary COVID-19 vaccination series were excluded. Analysis periods were determined based on U.S. variant proportion estimates. Rate denominators were calculated from vaccine administration data, with numbers of unvaccinated persons estimated by subtracting numbers of persons vaccinated with at least a primary series and persons with an incomplete primary series from 2019 U.S. intercensal population estimates.^¶¶^ A continuity correction assumed that ≥5% of each age group and jurisdiction would always be unvaccinated (i.e., ≤95% vaccination coverage).*** Average weekly incidence and mortality were calculated during each period and stratified by age group (12–17, 18–49, 50–64, 65–79 and ≥80 years) and vaccination status; overall rates were age-standardized using the 2000 U.S. Census Bureau standard population.^†††^ Two sets of analyses of incidence and mortality rates overall (24 jurisdictions) and by time since last monovalent or bivalent booster vaccination (23 jurisdictions) were conducted. Overall and strata-specific RRs were calculated by dividing rates among unvaccinated persons by rates among vaccinated persons; after detrending the underlying linear changes in rates, 95% CIs were calculated from the remaining variation in observed weekly rates^§§§^ (*8*,*9*). SAS (version 9.4; SAS Institute) and R (version 4.1.2; R Foundation) were used to conduct all analyses. This activity was reviewed by CDC and was conducted consistent with applicable federal law and CDC policy.^¶¶¶^

Among persons aged ≥12 years, a total of 21,296,326 COVID-19 cases and 115,078 associated deaths were reported during October 3, 2021–December 24, 2022, and October 3, 2021–December 3, 2022, respectively, from 24 U.S. jurisdictions (Table). Average weekly age-standardized incidence and mortality (cases and deaths per 100,000 population aged ≥12 years) increased substantially during the Omicron BA.1 period and to a lesser extent during the early BA.4/BA.5 period (Figure 1). During all periods, average weekly age-standardized incidence and mortality were consistently higher among unvaccinated persons (ranges = 216.1–1,256.0 and 1.6–15.8, respectively) than among monovalent-only vaccine recipients (ranges = 86.4–487.7 and 0.3–1.4, respectively); average weekly incidence and mortality during the late BA.4/BA.5 period were lowest among bivalent booster recipients (78.5 and 0.1, respectively).

**TABLE:** Average weekly incidence,* mortality rates,^†^ and rate ratios for unvaccinated compared with vaccinated persons, by age group, variant period,^§^ and receipt of bivalent booster doses — 24 U.S. jurisdictions,^¶^ October 2021–December 2022**

Dates (predominant variant)	Age group, yrs											
	12–17		18–49		50–64		65–79		≥80		All ages ≥12 yrs (age-standardized)	
	No.	Incidence	No.	Incidence	No.	Incidence	No.	Incidence	No.	Incidence	No.	Incidence
Cases*												
**Oct 3–Dec 18, 2021 (Delta)**												
Unvaccinated	238,129	384.9	1,101,981	443.9	320,532	473.5	135,567	729.8	36,528	457.9	**1,832,737**	**475.8**
Vaccinated	43,715	71.4	587,110	137.6	270,337	113.3	146,740	82.2	48,377	93.9	**1,096,279**	**118.3**
RR (95% CI)††	—	5.4 (2.7–10.7)	—	3.2 (1.9–5.3)	—	4.2 (3.2–5.5)	—	8.9 (7.1–11.1)	—	4.9 (4.0–6.0)	**—**	**4.0 (2.9–5.6)**
**Dec 19, 2021–Mar 19, 2022 (Omicron BA.1)**												
Unvaccinated	572,012	870.9	3,170,926	1,241.3	834,743	1,261.0	337,174	1,704.0	101,276	1,182.6	**5,016,131**	**1,256.0**
Vaccinated	426,833	507.2	3,202,802	572.9	1,230,476	405.0	581,903	258.2	168,564	259.6	**5,610,578**	**487.7**
RR (95% CI)††	—	1.7 (1.0–2.9)	—	2.2 (1.1–4.5)	—	3.1 (1.5–6.4)	—	6.6 (3.6–12.1)	—	4.6 (2.7–7.7)	**—**	**2.6 (1.6–4.1)**
**Mar 20–Jun 25, 2022 (Omicron BA.2)**												
Unvaccinated	65,272	98.7	546,933	212.5	170,798	252.4	90,598	442.0	33,135	375.6	**906,736**	**240.2**
Vaccinated	78,139	80.6	904,179	144.8	427,853	127.8	275,413	111.1	95,296	133.3	**1,780,880**	**130.8**
RR (95% CI)††	—	1.2 (0.9–1.6)	—	1.5 (1.3–1.6)	—	2.0 (1.8–2.2)	—	4.0 (3.6–4.4)	—	2.8 (2.5–3.1)	**—**	**1.8 (1.7–2.0)**
**Jun 26–Sep 17, 2022 (early Omicron BA.4/BA.5)**												
Unvaccinated	106,120	193.5	761,559	356.7	241,386	429.1	138,059	812.4	49,447	685.1	**1,296,571**	**417.3**
Vaccinated	69,144	81.7	863,788	160.0	471,681	162.9	350,412	162.8	128,081	205.9	**1,883,106**	**154.5**
RR (95% CI)††	—	2.4 (1.2–4.5)	—	2.2 (2.1–2.4)	—	2.6 (2.5–2.8)	—	5.0 (4.7–5.3)	—	3.3 (3.2–3.5)	**—**	**2.7 (2.6–2.8)**
**Sep 18–Dec 24, 2022 (late Omicron BA.4/BA.5)**												
Unvaccinated	46,384	74.0	377,142	155.3	146,444	228.4	100,317	511.1	46,026	554.4	**716,313**	**216.1**
Monovalent vaccine only	39,360	41.2	481,552	80.2	298,532	97.5	230,382	110.4	107,169	174.4	**1,156,995**	**86.4**
RR (95% CI)††	—	1.8 (1.2–2.8)	—	1.9 (1.5–2.5)	—	2.3 (1.9–2.9)	—	4.6 (3.8–5.6)	—	3.2 (2.6–4.0)	**—**	**2.5 (2.2–2.9)**
Bivalent booster	1,112	25.9	30,424	79.6	29,589	85.2	45,139	88.4	21,253	139.6	**127,517**	**78.5**
Bivalent booster RR	—	2.9 (1.8–4.4)	—	2.0 (1.5–2.5)	—	2.7 (2.2–3.3)	—	5.8 (4.8–6.9)	—	4.0 (3.2–5.0)	**—**	**2.8 (2.4–3.1)**
**Deaths^†^**												
**Oct 3–Dec 18, 2021 (Delta)**												
Unvaccinated	23	0.04	3,072	1.3	7,202	11.7	9,470	55.0	5,533	77.6	**25,300**	**12.2**
Vaccinated	2	0.004	232	0.1	1,111	0.5	3,279	2.1	4,041	9.3	**8,665**	**0.7**
RR (95% CI)††	—	10.8 (0.2–578.5)	—	20.9 (13.0–33.5)	—	21.4 (16.0–28.6)	—	25.8 (20.6–32.4)	—	8.3 (6.4–10.9)	**—**	**16.2 (14.1–18.7)**
**Dec 19, 2021–Mar 19, 2022 (Omicron BA.1)**												
Unvaccinated	25	0.04	2,495	1.0	6,917	11.3	12,836	70.5	10,220	131.0	**32,493**	**15.8**
Vaccinated	7	0.01	635	0.1	2,828	1.1	7,748	4.0	8,994	16.3	**20,212**	**1.4**
RR (95% CI)††	—	4.3 (0.5–41.2)	—	7.8 (5.0–12.1)	—	10.5 (7.6–14.4)	—	17.8 (13.2–24.0)	—	8.0 (5.5–11.7)	**—**	**11.5 (9.4–14.1)**
**Mar 20–Jun 25, 2022 (Omicron BA.2)**												
Unvaccinated	9	0.01	245	0.1	514	0.8	1,144	6.1	1,418	17.5	**3,330**	**1.6**
Vaccinated	2	0.002	133	0.02	501	0.2	1,503	0.7	2,700	4.4	**4,839**	**0.3**
RR (95% CI)††	—	6.2 (0.04–935.3)	—	4.0 (1.6–10.0)	—	4.7 (3.5–6.4)	—	8.7 (6.6–11.5)	—	4.0 (3.4–4.6)	**—**	**5.3 (4.6–6.0)**
**Jun 26–Sep 17, 2022 (early Omicron BA.4/BA.5)**												
Unvaccinated	12	0.02	332	0.2	790	1.5	1,725	11.1	2,154	32.4	**5,013**	**2.9**
Vaccinated	1	0.001	223	0.05	760	0.3	2,665	1.4	4,039	7.6	**7,688**	**0.5**
RR (95% CI)††	—	17.4 (0.03–9,462.7)	—	3.4 (1.9–5.9)	—	5.0 (3.7–6.6)	—	7.8 (6.9–8.9)	—	4.3 (3.6–5.0)	**—**	**5.3 (4.8–5.9)**
**Sep 18–Dec 3, 2022 (late Omicron BA.4/BA.5)**												
Unvaccinated	3	0.01	165	0.1	387	0.8	1,076	7.6	1,410	23.4	**3,041**	**2.0**
Monovalent vaccine only	0	0	81	0.02	397	0.2	1,369	0.9	2,358	5.5	**4,205**	**0.4**
RR (95% CI)††	—	—	—	4.5 (2.3–9.1)	—	4.4 (2.6–7.4)	—	8.3 (6.4–10.7)	—	4.2 (3.7–4.9)	**—**	**5.4 (4.8–6.1)**
Bivalent booster	0	0	1	0.005	12	0.1	91	0.3	188	2.3	**292**	**0.1**
Bivalent booster RR (95% CI)††	—	—	—	18.8 (0.8–467.9)	—	12.8 (2.7–61.5)	—	23.7 (12.6–44.7)	—	10.3 (7.0–15.2)	—	**14.1 (10.1–19.6)**

**Abbreviation**: RR = rate ratio.

* Cases per 100,000 persons aged ≥12 years. COVID-19 cases among unvaccinated persons and persons vaccinated with a primary series with or without a monovalent or bivalent booster dose were defined as previously described (https://www.cdc.gov/coronavirus/2019-ncov/php/hd-breakthrough.html). Cases with primary series or a monovalent booster were combined in the “vaccinated only with monovalent vaccines” category. Cases were excluded among persons who received ≥1 Food and Drug Administration–authorized vaccine dose but did not complete a primary series ≥14 days before the positive specimen collection date.

^†^ Deaths per 100,000 persons aged ≥12 years. A COVID-19–associated death occurred in a person with a documented COVID-19 diagnosis who died, and whose report local health authorities reviewed to make that determination (e.g., using vital records, public health investigation, or other data sources). Per national guidance, this group includes persons whose death certificate lists COVID-19 or SARS-CoV-2 as an underlying cause of death or a significant condition contributing to death. COVID-19 mortality by vaccination status is reported based on COVID-19 test date, not the date the patient died.

^§^ Analysis periods were categorized based on variant predominance (defined as accounting for >50% of sequenced isolates): Delta, October 3–December 18, 2021; Omicron BA.1, December 19, 2021–March 19, 2022; Omicron BA.2, March 20–June 25, 2022; early Omicron BA.4/BA.5, June 26–September 17, 2022; and late Omicron BA.4/BA.5 (only period in which bivalent boosters were recommended), September 18–December 24, 2022

^¶^ These 24 jurisdictions represent 52% of the overall U.S. population and were included in this analysis: Alabama, Arizona, Arkansas, Colorado, District of Columbia, Georgia, Idaho, Indiana, Kansas, Kentucky, Louisiana, Massachusetts, Michigan, Minnesota, Nebraska, New Jersey, New Mexico, New York, North Carolina, Tennessee, Texas, Utah, Washington, and West Virginia; New York did not provide mortality data

** Date range for average weekly incidence is October 3, 2021–December 24, 2022; date range for mortality rates is October 3, 2021–December 3, 2022.

^††^ 95% CIs were calculated after detrending underlying linear changes in weekly rates using piecewise linear regression. Each 95% CI represents the remaining variation in observed weekly rates and resulting RRs. The number of observations informing each 95% CI reflects the number of weeks per period: Delta (11), Omicron BA.1 (13), Omicron BA.2 (14), early Omicron BA.4/BA.5 (12), and late Omicron BA.4/BA.5 (14).

**FIGURE 1:**
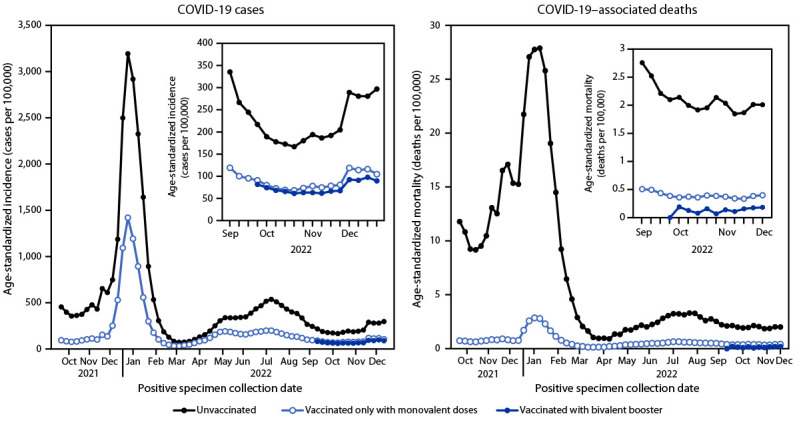
Age-standardized weekly COVID-19 incidence* and COVID-19–associated mortality rates,† by vaccination status and receipt of a bivalent booster dose^§^ — 24 U.S. jurisdictions,^¶^ October 2021–December 2022** * Cases per 100,000 persons aged ≥12 years. COVID-19 cases among unvaccinated persons and persons vaccinated with a primary series with or without a monovalent or bivalent booster dose were defined as previously described (https://www.cdc.gov/coronavirus/2019-ncov/php/hd-breakthrough.html). Cases with primary series or a monovalent booster were combined in the “vaccinated only with monovalent vaccines” category. Cases were excluded among persons who received ≥1 Food and Drug Administration–authorized vaccine dose but did not complete a primary series ≥14 days before the positive specimen collection date. ^†^ Deaths per 100,000 persons aged ≥12 years. A COVID-19–associated death occurred in a person with a documented COVID-19 diagnosis who died, and whose report local health authorities reviewed to make that determination (e.g., using vital records, public health investigation, or other data sources). Per national guidance, this group should include persons whose death certificate lists COVID-19 or SARS-CoV-2 as an underlying cause or a significant condition contributing to death. Rates of COVID-19 deaths by vaccination status are reported based on when the patient was tested for COVID-19, not the date the patient died. ^§^ Bivalent boosters were recommended during September 1–December 24, 2022. Based on case definitions, a case after vaccination occurred in a person ≥14 days postvaccination. ^¶^ These 24 jurisdictions represent 52% of the overall U.S. population and were included in this analysis: Alabama, Arizona, Arkansas, Colorado, District of Columbia, Georgia, Idaho, Indiana, Kansas, Kentucky, Louisiana, Massachusetts, Michigan, Minnesota, Nebraska, New Jersey, New Mexico, New York, North Carolina, Tennessee, Texas, Utah, Washington, and West Virginia; New York did not provide mortality data. ** Date range for age-standardized weekly COVID-19 incidence is October 3, 2021–December 24, 2022; date range for COVID-19–associated mortality rates is October 3, 2021–December 3, 2022.

Overall, age-standardized case RRs (unvaccinated persons compared with monovalent-only vaccine recipients) declined from 4.0 during the Delta period to 2.6 during the Omicron BA.1 and 1.8 during the Omicron BA.2 periods, before increasing to 2.7 in the early BA.4/BA.5 period. Overall case RRs (unvaccinated persons compared with bivalent booster recipients) were slightly higher (2.8) than were those for monovalent-only vaccine recipients (2.5) during the late BA.4/BA.5 period. Average, age-standardized mortality RRs in monovalent-only vaccine recipients decreased from the Delta (16.2) to BA.1 (11.5) period, then stabilized during the BA.2 (5.3), early BA.4/BA.5 (5.3), and late BA.4/BA.5 (5.4) periods. Overall mortality rates among unvaccinated persons were 14.1 times the rates among bivalent vaccine recipients; mortality rates among monovalent-only vaccine recipients were 2.6 times the rates among bivalent vaccine recipients during the late BA.4/BA.5 period. Compared with unvaccinated persons, protection among bivalent booster recipients aged 65–79 years (RR = 23.7), and ≥80 years (10.3) was significantly higher than was protection among monovalent booster recipients aged 65–79 years (8.3) and ≥80 years (4.2).

In stratified comparisons of unvaccinated persons and vaccinated persons who had received a monovalent booster dose 2 weeks–2 months earlier, progressive declines in case RRs were more pronounced between the Delta (7.0) and BA.1 (3.4), BA.2 (2.4), early BA.4/BA.5 (2.8), and late BA.4/BA.5 (2.8) periods; the case RR at 2 weeks–2 months after a bivalent booster (2.8) was the same during the late BA.4/BA.5 period (Figure 2) (Supplementary Table, https://stacks.cdc.gov/view/cdc/124201).**** A similar reduction in the case RR was observed for both the monovalent booster at 3–5 months (2.0) and the bivalent booster at 3 months (1.7) after vaccination during the late BA.4/BA.5 period. Mortality RRs for unvaccinated persons compared with persons who received a monovalent booster dose 2 weeks–2 months earlier declined from 50.7 during the Delta period to 21.4 during the BA.1 period, 7.9 during the BA.2 period, and 7.4 during the early BA.4/BA.5 period, representing a reduction in crude vaccine effectiveness (VE) from 98% (Delta) to 87% (BA.4/BA.5).^††††^ During the early BA.4/BA.5 period, mortality RRs remained stable 3–5 months after receiving a monovalent booster dose (7.2) but declined to 4.6 at 6–8 months, 4.5 at 9–11 months, and 2.5 at ≥12 months. In contrast, bivalent boosters received in the preceding 2 weeks–2 months during the late BA.4/BA.5 period provided enhanced protection against death (mortality RR = 15.2 in unvaccinated persons versus bivalent booster recipients), representing a crude VE of 93%. A subset analysis of the Omicron BA.5, BA.4, and BA.2-related variant period (November 6–December 24, 2022) yielded similar results.

**FIGURE 2:**
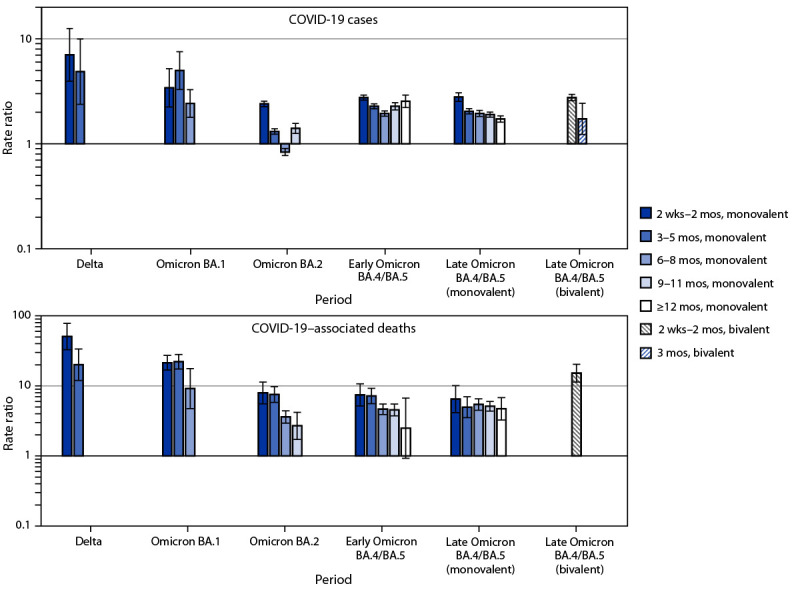
Age-standardized average weekly case* and mortality^†^ rate ratios with 95%CIs^§^ in unvaccinated persons compared with booster dose recipients, by variant period^¶^ and time since receipt of last booster dose** — 23 U.S. jurisdictions,^††^ October 2021–December 2022^§§^ * Cases per 100,000 persons aged ≥12 years. COVID-19 cases among unvaccinated persons and persons vaccinated with a primary series with or without a monovalent or bivalent booster dose were defined as previously described (https://www.cdc.gov/coronavirus/2019-ncov/php/hd-breakthrough.html). Cases were excluded in persons who only completed a primary series or who received ≥1 Food and Drug Administration–authorized vaccine dose but did not complete a primary series ≥14 days prior to the positive specimen collection date. ^†^ A COVID-19–associated death occurred in a person with a documented COVID-19 diagnosis who died, and whose report local health authorities reviewed to make that determination (e.g., using vital records, public health investigation, or other data sources). Per national guidance, this group should include persons whose death certificate lists COVID-19 or SARS-CoV-2 as an underlying cause or a significant condition contributing to death. Rates of COVID-19 deaths by vaccination status are reported based on when the patient was tested for COVID-19, not the date the patient died. ^§^ 95% CIs were calculated after detrending underlying linear changes in weekly rates using piecewise linear regression. Each 95% CI represents the remaining variation in observed weekly rates and resulting rate ratios. The number of observations leading to each 95% CI reflects the number of weeks per period: Delta (11), Omicron BA.1 (13), Omicron BA.2 (14), early Omicron BA.4/BA.5 (12), and late Omicron BA.4/BA.5 (14). ^¶^ Analysis periods were categorized based on variant predominance (defined as accounting for >50% of sequenced lineages): Delta, October 3–December 18, 2021; Omicron BA.1, December 19, 2021–March 19, 2022; Omicron BA.2, March 20–June 25, 2022; early Omicron BA.4/BA.5, June 26–September 17, 2022; and late Omicron BA.4/BA.5 (only period where bivalent boosters were recommended), September 18–December 24, 2022. ** Time since last monovalent booster categories was restricted to outcomes occurring during eligible weeks based on the timing of the first booster recommendation for adults aged ≥65 years and adults aged ≥18 years in high-risk groups on September 24, 2021: 2 weeks–2 months (starting October 3, 2021); 3–5 months (starting November 13, 2021); 6–8 months (starting February 13, 2022); 9–11 months (starting May 15, 2022); ≥12 months (starting August 14, 2022). For persons aged 12–17 years, boosters were recommended on January 5, 2022; data are included the week starting January 16, 2022. Bivalent boosters were included for the period starting September 18, 2022, and for categories of 2 weeks–2 months and 3 months after receipt of a booster for cases and 2 weeks–2 months after receipt of a booster for deaths. Unvaccinated persons are compared to vaccinated persons for the same time frame in each category. The median interval in the 2 weeks–2 months since vaccination period was longer for persons with monovalent boosters during early (60 days) and late (70 days) BA.4/BA.5 periods than for those who received bivalent boosters (47 days). The median interval among persons who received a monovalent booster 3–5 months earlier was 131 and 191 days, respectively, during early and late BA.4/BA.5 periods; among those who received bivalent boosters 3 months earlier, the median interval was 95 days. ^††^ These 23 jurisdictions represent 50% of the overall U.S. population and were included in this analysis: Alabama, Arizona, Arkansas, Colorado, District of Columbia, Georgia, Idaho, Indiana, Kansas, Kentucky, Louisiana, Michigan, Minnesota, Nebraska, New Jersey, New Mexico, New York, North Carolina, Tennessee, Texas, Utah, Washington, and West Virginia; New York did not provide mortality data. ^§§ ^Date range for age-standardized average weekly case rate ratio is October 3, 2021–December 24, 2022; date range for mortality rate ratio is October 3, 2021–December 3, 2022.

## Discussion

This multijurisdictional report of COVID-19 case and mortality rates included two sets of analyses with different comparisons by vaccination status. In the first, overall rates among unvaccinated persons were compared to rates in persons with only monovalent doses or bivalent boosters. Receipt of bivalent booster added protection against infection and death for circulating Omicron BA.4/BA.5 sublineages. When stratifying by time since vaccination for the second analysis, comparisons during the late BA.4/BA.5 period of monovalent and bivalent boosters found that bivalent boosters restored protection against mortality and provided similar protection against infection at 2 weeks through 2 months. Although long-term protection could not yet be assessed, evidence of waning protection against infection 3 months after bivalent booster dose receipt was observed. This study supports previous findings of protection afforded by bivalent vaccines against infection and medically attended illness during BA.4/BA.5 predominance (*5*–*7*) and provides additional evidence of enhanced protection against COVID-19–associated mortality. To date, however, bivalent booster coverage has been low (17.5% among persons aged ≥12 years).^§§§§^

During the early BA.4/BA.5 period, waning protection against COVID-19–associated death was observed ≥6 months after receipt of monovalent boosters, although decreases were not always statistically significant. Findings were similar to those reported in a 2021 study on waning immunity from primary COVID-19 vaccination during the Delta period (*4*). Patterns of waning protection against COVID-19–associated death after receiving a monovalent booster were less apparent during the late BA.4/BA.5 period; this might be related to smaller sample sizes and potential boosts to immunity over time resulting from recent infections or receipt of boosters that were not matched to existing vaccination records. Well-controlled VE studies conducted during the BA.4/BA.5 period have shown waning protection of monovalent doses against hospitalization starting at 4 months, with incremental benefits of bivalent boosters with increasing time since the last monovalent dose^¶¶¶¶^ (*6*,*7*).

The findings in this report are subject to at least six limitations. First, authorizations for monovalent and bivalent boosters were not concurrent; the median time after vaccination was longer for persons who received monovalent boosters than for those who received bivalent boosters, which limits direct comparability. Second, distinguishing monovalent boosters from additional primary doses administered to immunocompromised persons was not possible, which could result in reduced RRs because of lower VE in this population. Third, this ecologic study could not adjust for important confounders that might contribute to rate differences, such as possible variations in infection-derived immunity, co-morbidities, and testing or prevention behaviors by age and vaccination status (*1*). Increased at-home test use has affected trends in case incidence more than trends in mortality over time (*1*); however, increases have been noted in COVID-19–associated deaths without laboratory-confirmation,***** which were not included in data reported by vaccination status, possibly reducing recent RRs. Fourth, national variant prevalence estimates were used, but variant prevalence differs by region. Fifth, misclassification of bivalent or monovalent boosters could influence RRs (*10*). Cases in bivalent booster recipients might have been preferentially identified because accounting for bivalent doses reported as first and second doses was possible, whereas distinguishing unlinked monovalent boosters from first or second doses was not possible. Finally, these data represent approximately one half of the U.S. population, and therefore, might not be generalizable.

This report presents evidence of the enhanced protection provided by bivalent COVID-19 boosters compared to monovalent vaccines against infection and death during the BA.4/BA.5 period and are consistent with other VE studies. Continued monitoring of the impact of emerging variants on VE against severe COVID-19 outcomes is needed. For the best protection against severe COVID-19, all persons should stay up to date with recommended COVID-19 vaccination, including receipt of a bivalent booster by eligible persons.

**Summary**

**What is already known about this topic?**

COVID-19 vaccine effectiveness decreased with waning of vaccine-derived immunity and emerging Omicron sublineages. An updated (bivalent) booster dose enhances protection against infection and medically attended illness, but protection against death has not been evaluated.

**What is added by this report?**

Bivalent booster recipients in 24 U.S. jurisdictions had slightly higher protection against infection and significantly higher protection against death than was observed for monovalent booster recipients or unvaccinated persons, especially among older adults.

**What are the implications for public health practice?**

Bivalent COVID-19 booster doses protected against infection and death during BA.4/BA.5 circulation. All eligible persons should get 1 bivalent booster dose ≥2 months after their COVID-19 primary series or last monovalent booster dose.

## Footnotes

*
https://www.cdc.gov/coronavirus/2019-ncov/vaccines/stay-up-to-date.html

† National weighted estimates of weekly proportions of infections attributed to SARS-CoV-2 variants are based on CDC analyses (https://covid.cdc.gov/covid-data-tracker/#variant-proportions). Analysis periods were categorized based on variant predominance (defined as those accounting for >50% of sequenced lineages): Delta, October 3–December 18, 2021; Omicron BA.1, December 19, 2021–March 19, 2022; Omicron BA.2, March 20–June 25, 2022; early Omicron BA.4/BA.5, June 26–September 17, 2022; and late Omicron BA.4/BA.5 (only period in which bivalent boosters were recommended), September 18–December 24, 2022. A subset analysis was performed for the Omicron BA.5-, BA.4-, and BA.2-related variant period (November 6–December 24, 2022).

§
https://covid.cdc.gov/covid-data-tracker/#covidnet-hospitalizations-vaccination

¶ The 24 jurisdictions included in this analysis represent 52% of the U.S. population: Alabama, Arizona, Arkansas, Colorado, District of Columbia, Georgia, Idaho, Indiana, Kansas, Kentucky, Louisiana, Massachusetts, Michigan, Minnesota, Nebraska, New Jersey, New Mexico, New York, North Carolina, Tennessee, Texas, Utah, Washington, and West Virginia. A subset of 23 jurisdictions (50% of the US population) was included in the time since last booster vaccination analysis; New York did not provide mortality data.

** A COVID-19 case (confirmed or probable) was defined as the detection of SARS-CoV-2 RNA or antigen in a respiratory specimen collected per the Council of State and Territorial Epidemiologists’ update to the standardized surveillance case definition and national notification for COVID-19 (https://cdn.ymaws.com/www.cste.org/resource/resmgr/ps/ps2022/22-ID-01_COVID19.pdf). Reinfections occurring after 90 days were counted as new cases, per current guidance.

†† A COVID-19–associated death occurred in a person with a documented COVID-19 diagnosis who died and whose report local health authorities reviewed to make that determination (e.g., using vital records, public health investigation, or other data sources). Per national guidance, this group should include persons whose death certificate lists COVID-19 or SARS-CoV-2 as an underlying cause or a significant condition contributing to death (https://preparedness.cste.org/wp-content/uploads/2022/12/CSTE-Revised-Classification-of-COVID-19-associated-Deaths.Final_.11.22.22.pdf). Rates of COVID-19 deaths by vaccination status are reported based on when specimens were tested for COVID-19, not the date the patient died.

§§ COVID-19 cases among unvaccinated persons and persons vaccinated with a primary series with or without a monovalent or bivalent booster dose were defined as previously described (https://www.cdc.gov/coronavirus/2019-ncov/php/hd-breakthrough.html). COVID-19 patients who had received a primary series or a monovalent booster were combined in the “vaccinated with monovalent vaccines only” category. Cases were excluded among persons who received ≥1 FDA-authorized vaccine dose but did not complete a primary series ≥14 days before the positive specimen collection date.

¶¶
https://www.census.gov/programs-surveys/popest/data/tables.2019.html

*** A continuity correction was applied to denominators by capping the percentage of population coverage at 95%. To do this, it was assumed that ≥5% of each age group would always be unvaccinated in each jurisdiction. Adding this correction ensures that there is always a reasonable denominator for the unvaccinated population and prevents incidence and death rates from growing unrealistically large because of potential overestimates of vaccination coverage.

††† Age-standardization was performed using the direct method with the year 2000 projected U.S. population, per the 1998 directive on adjusting mortality data from the Secretary of the U.S. Department of Health and Human Services (https://www.cdc.gov/nchs/data/statnt/statnt20.pdf).

§§§ 95% CIs were calculated after detrending underlying linear changes in weekly rates using piecewise linear regression. Each 95% CI represents the remaining variation in observed weekly rates and resulting RRs. The number of observations leading to each 95% CI reflects the number of weeks per period: Delta (11), Omicron BA.1 (13), Omicron BA.2 (14), early Omicron BA.4/BA.5 (12), and late Omicron BA.4/BA.5 (14).

¶¶¶ 45 C.F.R. part 46.102(l)(2), 21 C.F.R. part 56; 42 U.S.C. Sect. 241(d); 5 U.S.C.0 Sect.552a; 44 U.S.C. Sect. 3501 et seq.

**** The median interval in the 2 weeks–2 months since vaccination period was longer for persons who received monovalent boosters during early (60 days) and late (70 days) BA.4/BA.5 periods than for those who received bivalent boosters (47 days). The median interval among persons who received a monovalent booster 3–5 months earlier was 131 and 144 days during early and late BA.4/BA.5 periods, respectively; among those who received bivalent boosters 3–5 months earlier, the median interval was 95 days.

†††† To interpret rate ratio changes, age-standardized crude vaccine effectiveness was estimated as (1 − [incidence in vaccinated / incidence in unvaccinated]) x 100%.

§§§§
https://covid.cdc.gov/covid-data-tracker/#vaccinations_vacc-total-admin-rate-total

¶¶¶¶
https://www.cdc.gov/vaccines/acip/meetings/downloads/slides-2022-09-01/04-covid-link-gelles-508.pdf

*****
https://www.cdc.gov/coronavirus/2019-ncov/science/data-review/hospitals.html
